# Chronic Dyspepsia in Older Children

**Published:** 1906-02-17

**Authors:** 


### Hospital Clinics.

### CHRONIC DYSPEPSIA IN OLDER CHILDREN.

In a recent lecture on this subject Dr. Thursfield [1] observes that of all the out-patients of two years old and upward attending the practice of a children's hospital, at least half are there on account of a failure in the proper assimilation of their food. This chronic dyspepsia is not in these later years of childhood of the same ominous significance as in the years of infancy, but though it does not directly kill, it is yet capable of stunting growth, reducing vitality, and inducing a very great degree of bodily distress.

The causes of chronic dyspepsia are varied, but a common one is to be found in a carious condition of the teeth. Mere decay of the teeth is not of itself a potent cause, but the neglect of the hygiene of the mouth which leads to the decay, and thus accentuates the original evil, is capable of exercising a very harmful effect upon the tissues and functions of the digestive tract. It is possible that the unhealthy condition of the teeth and gums results in a certain number of cases in a chronic poisoning of the lymphatic tissues about the oral cavity, and thus induces the chronic hypertrophy of the pharyngeal and post-nasal glandular tissues, which is so common an associate of chronic dyspepsia. But in a large number of cases the hypertrophic condition of these tissues must be attributed to other less definite causes.

In a vast majority of all cases of chronic dyspepsia at this time of life, the cause is to be found in dietetic errors. Among the children of the well-to-do the error is commonly in the direction of excess, both in the matter of quantity and quality: that is to say the diet is too nutritious and improperly regulated. Among hospital patients, on the other hand, the fault is usually an excessive proportion of carbohydrate, not infrequently associated with an absolute deficiency in quantity. Apart, however, from these gross errors in dietetics there are children whose digestions are not normal, children who have to be fed abnormally, simply because their digestive apparatus is, for the time being at least, anomalous.

The symptoms of the condition under discussion are manifold, but one symptom, and one alone, is constant. This is the failure of the affected child to "get on." The child is usually thin and wasted, with a dry harsh skin, round shoulders, a flattened chest and a protuberant belly: he is anæmic, and easily fatigued both bodily and mentally; he is drowsy by day and restless at night. His appetite is capricious, and the action of his bowels very uncertain, diarrhœa alternating with constipation. In either event it is common to find an excess of mucus in the stools. Children in such a state of health are very prone to fainting fits of doubtful meaning. "The child does not lose consciousness, but quite suddenly turns white, is collapsed for some seconds and then slowly regains his natural colour." These attacks do not indicate any lesion of the heart, and are not of much significance except as reflecting the general condition. Dr. Thursfield points out that occasionally such fainting-fits may be, in fact, expressions of minor epilepsy, especially when associated with recurring and acute attacks of abdominal pain.

Constipation is more common than diarrhœa. The stools are large, offensive, and consist of scybalous masses, often containing undigested food. This constipation alternates with diarrhœa, and both conditions are frequently accompanied, as has been said, with the passage of mucus—spoken of by the mothers as "slime" or "jelly." This circumstance led Dr. Eustace Smith to christen the condition "Mucous disease," but in Dr. Thursfield's experience the absence of mucus is no rarity even in well-marked cases of chronic intestinal dyspepsia. The diarrhœa, when it occurs, is often of the "lienteric" variety. That is to say that the ingestion of food invariably leads to an immediate loose motion. This indicates an excessive irritability of the nervous and muscular mechanism of the intestine and is usually amenable to small doses of Fowler's solution.

Other less common symptoms are occasional fever, and a persistent irritating cough, neither of which are of much intrinsic importance, but gain dignity from the alarm which both are capable of exciting in the minds of mothers.

The diagnosis is not difficult if the condition be borne in mind. The most common confusion, both on the part of the parent and the doctor, is to mistake chronic intestinal dyspepsia for tuberculous disease of the lungs. In either case there is wasting, cough, liabilty to sweat on little provocation, lassitude, and general irritabiity. Careful physical examination of the thorax will go far towards setting the matter to rest. Dr. Thursfield mentions as a very valuable sign of early tuberculosis of the lungs, limitation of movement of the sub-clavicular area on one side, or its lagging behind the other. This is best estimated by standing behind the patient with the forefingers of the two hands placed upon the clavicles. Even where a suspicion of apical lesion is detected it is recommended that sentence should not be pronounced until a second examination has been made at an interval of some two weeks, suitable anti-dyspeptic treatment being initiated in the interval.

Prognosis is good, but in a minority of cases it is necessary to persevere in the rigorous supervision of the diet for a length of time. The only circumstance which is likely to cause real anxiety is the intercurrence of some acute disease while the vitality of the system is low.

The general principles of treatment embrace attention to faulty conditions of the teeth and tonsils if necessary; secondly, supervision of hygienic matters such as ventilation, clothing, regularity of habits, sleep and exercise; and lastly extreme care in the regulation of the diet and functions of the alimentary canal. The most important elements in the dietetic *régime* are the elimination of irregular and injudicious meals. The first essential, therefore, is abstention from sweetmeats, rich cakes, sweet biscuits, chocolates, and similar dainties, not only between meals, but altogether. The meals should be regular, not more than four a day, and nothing should be given between meals. In addition to the confectionery mentioned above, it is well to forbid new bread, potatoes, pickles, fried fish, and coarse vegetables. The diet most suitable to these children is one consisting of milk and bland dishes made with milk, such as rice and other milk puddings. If, however, the indigestion be severe, it may be necessary to exclude all varieties of carbohydrate for a while, and rely only upon milk, eggs, meat, and fish, with a little cooked fruit and toast or stale bread.

It is wise to supplement the dietetic treatment with the administration of bitters or tonics; an alkali, for instance, with a little tincture of nux vomica, or iron and arsenic; while in the early stages of treatment the employment of mild aperients seems to advance the cure even though there be no absolute call for them. For this purpose the most effective drugs are calomel, rhubarb, senna or salts.

When the bowels are acting regularly, when the tongue is clean, and appetite is returning, iron and cod-liver oil or one of its substitutes may be employed with advantage, and a recalcitrant case may on occasion be made quite amenable to treatment by a visit to the country or the sea-side.

[1] Clin. Jour., Jan. 31, 1906.

